# Paraganglion, a pitfall in diagnosis after regular cholecystectomy

**DOI:** 10.1016/j.ijscr.2019.10.042

**Published:** 2019-10-28

**Authors:** Bartholomeus J.G.A. Corten, Wouter K.G. Leclercq, M. Wouter Dercksen, Wilhelmus T. van den Broek, Peter H. van Zwam, Cees H. Dejong, Gerrit D. Slooter

**Affiliations:** a Department of Surgery, Máxima Medical Center, Veldhoven, the Netherlands; b Department of Internal Medicine, Máxima Medical Center, Veldhoven, the Netherlands; c Department of Surgery, St, Anna Hospital, Geldrop, the Netherlands; d Department of Pathology, PAMM Laboratory for Pathology and Medical Microbiology, Eindhoven, the Netherlands; e Department of Surgery, Maastricht University Medical Centre, Maastricht, the Netherlands

**Keywords:** NET, neuroendocrine tumour, MEN1, Multiple Endocrine Neoplasia Type 1, GBC, gallbladder cancer, CT, computed tomography, PETCT, positron emission tomography–computed tomography, Ga-DOTATOC-PET, (68)Ga-DOTA(0)-Phe(1)-Tyr(3)-octreotide Positron Emission Tomography Computed Tomography, WHO, World Health Organization, NEC, neuroendocrine carcinoma, MINEN, mixed neuroendocrine-nonneuroendocrien neoplasms, Hpf, high power fields, AJCC, American Joint Committee on Cancer, TNM, tumor, nodes, and metastases, PRRT, Peptide Receptor Radionuclide Therapy, HE, hematoxylin-eosin staining, Gallbladder cancer, Neuroendocrine neoplasm, Neuroendocrine tumour, Paraganglion

## Abstract

**Introduction:**

Neuroendocrine neoplasm of the gallbladder is an extremely uncommon diagnosis. We present a case of a benign gallbladder paraganglion that was initially incorrectly diagnosed as a neuroendocrine tumour (NET).

**Presentation of case:**

A 27-year-old female with symptomatic gallstone disease underwent an uncomplicated laparoscopic cholecystectomy. Routine histopathologic examination suggested the presence of a small adventitial NET. However, histopathological revision was performed by our pathologist because of regional gallbladder carcinoma (GBC) treatment evaluation. The revision demonstrated the presence of a normal paraganglion, a preexistent structure that is only rarely encountered during routine histopathologic examination of the gallbladder.

**Discussion:**

Neuroendocrine neoplasms of the gallbladder are extremely rare. Treatment varies from a simple cholecystectomy to extensive surgical resections. Chemotherapy is usually reserved for metastatic disease. In contrast, a gallbladder paraganglion is a benign entity not requiring additional treatment.

**Conclusion:**

A neuroendocrine neoplasm of the gallbladder may closely resemble a benign paraganglion. If a NET is suspected, the clinician should be aware of the histopathologic mimicry of a paraganglion prior to initiating additional treatments.

## Highlights

• Neuroendocrine neoplasms are a rarity after cholecystectomy and current literature is scarce.
• Paraganglion of the gallbladder is an incidental benign finding.
• A paraganglion can mimic the histopathologic appearance of neuroendocrine tumours.

## 1 Introduction

A neuroendocrine neoplasm of the gallbladder is an extremely uncommon diagnosis. Physicians may be confronted with a gallbladder neuroendocrine neoplasm as a coincidental histopathological finding following a standard cholecystectomy for benign gallstone disease. In general, an up to 2.8 % incidence of gallbladder malignancy is demonstrated following an elective cholecystectomy, and just 2 % of these 2.8 % are neuroendocrine neoplasms [1]. We present a patient who was initially diagnosed with a neuroendocrine tumour (NET) after cholecystectomy. A second analysis was performed years later because of regional gallbladder carcinoma (GBC) treatment evaluation. This revision however concluded that a paraganglion was the true diagnosis. This is an even more uncommon outcome after a routine cholecystectomy. The aim of this case report is to increase awareness regarding the differential diagnosis of unusual pathological findings in gallbladder tissue. This case report has been reported in line with the SCARE criteria [2].

## 2 Case report

In 2007, a previously healthy 27-year-old Caucasian female was referred to the surgical outpatient clinic for analysis of abdominal pain. The upper right quadrant pain had started a few months earlier and was intermittently present up to one hour following food ingestion. Jaundice, fever, vomiting, melena or altered bowel habits were absent. The family history for cancer or MEN-1 disease (Multiple Endocrine Neoplasia Type 1) was unknown

Physical examination only revealed mild pain following deep upper quadrant palpation without signs of peritonitis. As gallbladder disease was likely, ultrasonography of the abdomen was advised that demonstrated cholecystolithiasis. The morphology of the gallbladder was deemed normal as were all other abdominal organs. The patient was planned for a laparoscopic cholecystectomy that was performed uneventfully a few months later.

**Fig. 1:**
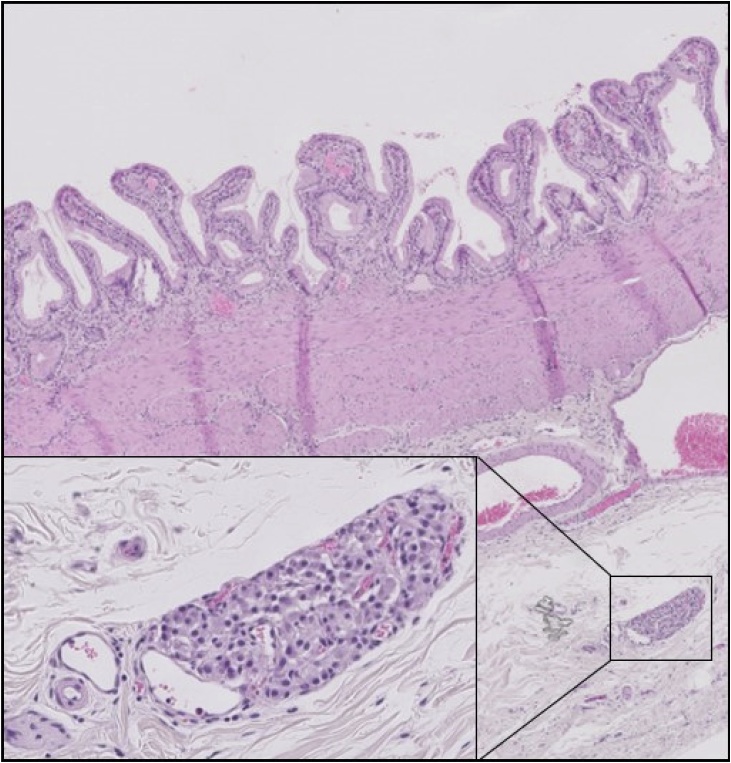
Hematoxylin-eosin staining (HE) showing part of the normal gallbladder wall. Adjacent to a blood vessel in the subserosal tissue a paraganglion (inset). (Hematoxylin-Eosin staining (HE)).

A routine histopathologic examination showed a 0,3 mm NET in the adventitia of the gallbladder with clear resection margins (Fig. 1). A subtype was not defined. The patient was not presented in our multidisciplinary oncology meeting. A yearly out-patient follow-up with focused echography of the liver during 5 years showed no recurrence of disease. Follow-up was stopped. A recent telephone contact assured that patient is currently disease-free and in excellent health. Surprisingly, histological revision of her gallbladder by our pathologist dictated by a regional GBC research initiative showed a paraganglion of the gallbladder instead of a NET.

## 3 Discussion

GBC is a rare form of cancer with a declining incidence in the Netherlands [3]. Nonetheless, it is number six on the list of gastrointestinal malignancies. Adenocarcinoma is the most common histologic type of GBC although adeno-squamous carcinoma, squamous cell carcinoma, and neuroendocrine neoplasms may also occur [4]. A neuroendocrine neoplasm is an epithelial malignant tumour with a predominant neuroendocrine differentiation as shown morphologically, immunohistochemically, and sometimes clinically (if they are secreting (neuro)endocrine compounds). A NET of the gallbladder is rare. It accounts for merely 0,2 - 0,5 % of all neuroendocrine neoplasms, and just 2 % of all malignant gallbladder tumours [[5], [6], [7], [8]].

The diagnosis of a gallbladder NET is difficult due to its rarity. In addition, presenting symptoms are generally non-speciﬁc including right upper quadrant abdominal pain, jaundice and weight loss. Most lesions are found after routine histology following cholecystectomy for symptomatic cholelithiasis. Fewer than 1 % of all NETs present with a carcinoid syndrome or rarely with a variety of other paraneoplastic syndromes [9].

These cases, patients are diagnosed in an advanced stage of the disease and survival varies depending on subtype. Radiological tests including ultrasonography, computed tomography scan (CT scan), magnetic resonance imaging and Positron emission tomography–computed tomography (PETCT) scans can reveal gallbladder masses indicative of gallbladder cancer. Ga-DOTATOC-PET scans ((68)Ga-DOTA(0)-Phe(1)-Tyr(3)-octreotide Positron Emission Tomography Computed Tomography) can distinguish between a neuroendocrine neoplasm and GBC, but are almost never performed preoperatively in case of suspicion of benign gallbladder disease. Therefore, it is almost impossible to clinically, radiologically, or intra-operatively distinguish neuroendocrine neoplasms from other subtypes of gallbladder carcinomas [10,11]. Pathological examination and immunohistochemical stainings such as chromogranin A, synaptophysin and Ki-67 are required for the diagnosis of a neuroendocrine neoplasm.

**Fig. 2:**
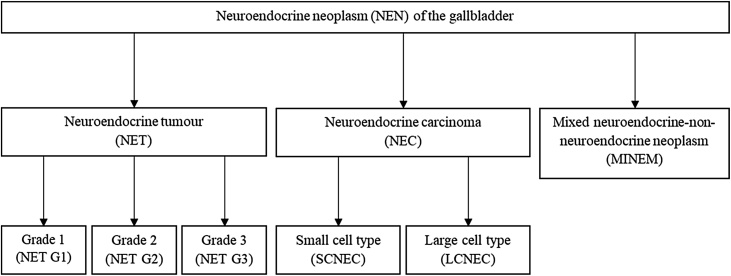
WHO classification of tumours of the digestive system. Subgroups include; NET G1, G2, G3; NEC; and mixed neuroendocrine-nonneuroendocrien neoplasms (MINEN).

Neuroendocrine neoplasms of the gallbladder are classified into three main histological categories according to the current World Health Organization (WHO) Classification of Tumours of the Digestive System (2019): neuroendocrine tumor (NET), neuroendocrine carcinoma (NEC), and mixed neuroendocrien-nonneuroendocrine neoplasms (MINEN) [4] (Fig. 2). Neuroendocrine neoplasms of the gallbladder do not have a separate staging system, unlike neuroendocrine neoplasms from other gastrointestinal sites. For staging, the American Joint Committee on Cancer (AJCC) Tumor, nodes, and metastases-staging (TNM) system is used.

NET are well-differentiated tumors divided into NET1, NET2 and NET3. Grading is mainly based on the assessment of proliferation. NET-G1 is characterized by a mitotic count of less than 2 mitoses per 10 high power fields (hpf) and/or a Ki-67 proliferation index of less than 3 %. NET-G2 is characterized by a mitotic count of 2–20 mitoses per 10 hpf and/or a Ki-67 proliferation index between 3 % and 20 %. NET-G3 have more than 20 mitoses/2 mm2 and a Ki-67 proliferation index of more than 20 %, but they are morphologically not poorly differentiated unlike NEC. NEC are further subdivided into small cell and large cell carcinoma based on cytonuclear features like the amount of cytoplasm and the presence of prominent nucleoli [4].

MINEN have a phenotype that is morphologically recognizable as neuroendocrine and also nonneuroendocrine epithelial (mostly adenocarcinoma) and is diagnosed only when both portions form more than 30 % of the tumor [4,12].

The first NET-G1 (previously known as carcinoid tumor) was described in 1888, while the first primary NET-G1 tumor of the gallbladder was reported by Joel in 1929 [13]. The most common locations of NET are the gastrointestinal tract (67 % of the cases) and bronchopulmonary tract (25 %) [14]. It is thought that NETs of the gallbladder originate from either metaplasia of gallbladder epithelium or from undifferentiated stem cells [10]. Neuroendocrine cells usually do not exist in a normal gallbladder. Neuroendocrine cells are located throughout the entire gut and were initially thought to arise from neural crest cells, but current insight suggests an origin from a multipotent stem cell [15]. However, the mechanisms that regulate the phenotypical expression and differentiation of cells of the diffuse endocrine-cell system are still poorly understood. NETs are, like other more frequented gallbladder tumours, usually associated with gallstones. The tumours can arise from the neck, fundus or the body of the gallbladder. Age of presentation can be as low as 20 years, but the average age of presentation is usually the 6th or 7th decade. Male-to-female ratio is 1:1.2 [14].

The surgical treatment of NETs of the gallbladder varies widely and is similar to other types of gallbladder cancer, ranging from cholecystectomy to extensive surgical resections, including regional lymph node dissection with possible hepatic lobectomy. Due to the rarity of the NETs, distinctive surgical strategy is missing for NETs in comparison to other types of gallbladder cancer. The therapeutic value of chemotherapy remains unclear, but is likely the best form of palliation of metastatic NET. Other palliative treatments like octreotide, Peptide Receptor Radionuclide Therapy (PRRT) or everolimus are reserved for metastatic NEC [16]. Additionally, the role of radiotherapy is unclear since in general NETs are, unlike NECs, insensitive to traditional radiotherapy [17].

The 5 year survival rate for NETs is versatile and mainly dependent on the subtype. Thus, NET grade I has a favorable prognosis compared to NET grade II or a NEC. Soga et al. found 5 year survival rates of 60.4 % and 21.3 % for NET grade I and NET grade II respectively [10,18].

Paraganglions of the gallbladder are in contrast a benign entity [19] and literature is scarce. Paraganglionic tissue is incidentally observed in the gallbladder specimen in the subserosal connective tissue. It is a relatively unknown outcome to many clinicians, with no clinical consequences. Only a handful of case reports of paraganglion of the gallbladder are described in the literature, although none of them report on the histopathologic mimicry of a neuroendocrine tumour and paraganglion [19,20]. Paraganglion tissue is merely a histopathological finding which can mimic a neuroendocrine neoplasm without any clinical relevance nor any relation to other diseases. Following an incidental finding of paraganglion of the gallbladder, no additional investigation nor follow-up is required. A revision of the histopathology is not necessary if the pathologist is certain of their findings. In conclusion, the incidental finding of the benign paraganglion finding remains inconsequential in contrast to NET or GBC.

Paraganglion should not be confused with another entity of the gallbladder know as a paraganglioma. These paraganglioma of the gallbladder arises from chief and sustentacular cells origination from the neural crest cells [21]. Paragangliomas can be functional or non-functional, and are rare extra-adrenal tumours of the paraganglia. Typical histopathologic findings include small island forming Zellballen nests and chromogranin and synaptophysin immunohistochemical staining for tumour cells. The diagnosis is usually incidental following a cholecystectomy, and no further treatment is required following a cholecystectomy.

In short, NETs of the gallbladder are an extremely uncommon form of the already rare entity of GBC and dependent on the subtype can show aggressive behaviour and poor prognosis. Patients present usually with symptoms of benign gallstone disease. NETs are clinically indistinguishable from other types of gallbladder cancer. Radical surgery is recommended for localised disease and may include lymphadenectomy combined with hepatic resection. Conversely, a paraganglion of the gallbladder is a benign condition which does not require additional surgery following a cholecystectomy.

## 4 Conclusion

We encountered a case of an otherwise healthy patient, with a unexpected diagnosis of the gallbladder. Initial diagnosis was a NET-grade 1 of the gallbladder. However, revision of the pathology years later, showed a benign paraganglion. With this case-report we would like to draw attention of clinicians to the histopathologic mimicry of neuroendocrine neoplasms and paraganglion tissue.

## Sources of funding

No funding to disclose.

## Ethical approval

Ethical approval is not needed for this case report.

## Consent

Informed consent was obtained for publication of this case report.

## Author contribution

Conception and design of study: BJGA Corten, WKG Leclercq.

Acquisition of data: BJGA Corten, W van den Broek, PH van Zwam.

Drafting the manuscript: BJGA Corten, WKG Leclecq, MW Dercksen, PH van Zwam.

Revising the manuscript critically for important intellectual content: BJGA Corten, WKG Leclercq, MW Dercksen, W van den Broek, PH van Zwam, C Dejong, GD Slooter.

## Registration of research studies

No registration of research studies.

## Guarantor

BJGA Corten, GD Slooter.

## Provenance and peer review

Not commissioned, externally peer-reviewed.

## Declaration of Competing Interest

The authors declare that they have no competing interests regarding the publication of this manuscript.
